# Isolation and Identification of Entomopathogenic Fungus GC23620 and Its Virulence and Control Efficacy Against *Gynaephora qinghaiensis* Larvae

**DOI:** 10.3390/biology15090678

**Published:** 2026-04-25

**Authors:** Zexi Lin, Siyu Liu, Youpeng Lai

**Affiliations:** Key Laboratory of Agricultural Integrated Pest Management of Qinghai Province, Qinghai Academy of Agricultural and Forestry Sciences, Qinghai University, Xining 810016, China; lzx97xx@163.com (Z.L.);

**Keywords:** *Gynaephora qinghaiensis*, entomopathogenic fungi, isolation and identification, pathogenicity, control efficacy on grassland, biological control

## Abstract

In June 2023, a larva of grassland caterpillar *Gynaephora qinghaiensis* naturally infected by an entomopathogenic fungus was collected from an alpine rangeland in Gangcha County, Haibei Tibetan Autonomous Prefecture, Qinghai Province. After laboratory isolation and cultivation, the pathogen was identified as *Beauveria bassiana* (designated as GC23620) based on morphological characteristics and ITS-rDNA sequence similarity analysis. The larvicidal efficacy of *B. bassiana* GC23620 against fourth-instar larvae of *G. qinghaiensis* were assessed using two inoculation methods in laboratory conditions. The infection process and pathogenicity were analyzed by simulation and parameter estimation using the Time–Dose–Mortality (TDM) model. The estimated parameters for the concentration effect of strain GC23620 (*β*) were 0.56 (leaf dipping method) and 0.30 (insect immersion method), respectively. After treatment with conidial suspensions (1.05 × 10^5^ to 1.05 × 10^9^ conidia/mL), the cumulative corrected mortalities were 72.73–100.00% (leaf dipping method) and 42.42–90.91% (insect immersion method) at 8 days after inoculation (DAI), and the median lethal doses (LD_50_) decreased to 1.74 × 10^3^ conidia/mL (leaf dipping method) and 1.85 × 10^4^ conidia/mL (insect immersion method), respectively, during the same post-inoculation period. After inoculation with conidial suspension under a concentration of 1.05 × 10^6^ conidia/mL, the median lethal times (LT_50_) were 2.40 (leaf dipping method) and 4.51 days (insect immersion method). A control efficacy of 84.27% was obtained for *G. qinghaiensis* larvae on grassland at 21 days post-treatment after spraying the fermentation solution with a low dose of 1.05 × 10^5^ conidia/mL. In conclusion, *B. bassiana* strain GC23620 exhibited high pathogenic activity against *G. qinghaiensis* larvae and has strong potential for the green control of grassland pests.

## Simple Summary

The grassland caterpillar *Gynaephora qinghaiensis* is a major pest on the Qinghai–Tibet Plateau, damaging alpine meadows and affecting livestock health. Traditional chemical control methods pose environmental risks, highlighting the need for sustainable alternatives. In this study, an entomopathogenic fungus (designated GC23620) was isolated from naturally infected *G. qinghaiensis* larvae in Qinghai Province and identified as *Beauveria bassiana*. Laboratory tests using leaf dipping and insect immersion methods demonstrated high virulence against fourth-instar larvae, with the leaf dipping method showing superior efficacy. Control efficacy on grassland confirmed that spraying a low-concentration suspension of GC23620 provided a control efficacy of 84.27% after 21 days. These findings suggest that *B. bassiana* strain GC23620 is a promising candidate for the biocontrol of *G. qinghaiensis*, supporting eco-friendly pest management in alpine grasslands.

## 1. Introduction

*Gynaephora qinghaiensis* (grassland caterpillar), also known as the red head black caterpillar, belongs to Lymantriidae family (Lepidoptera) and *Gynaephora* in general. To date, a total of 15 species of this genus have been recorded worldwide, of which eight are endemic on the Qinghai–Tibetan Plateau (3000–5000 masl), where they have seriously damaged alpine meadow, montane meadow, and meadow steppe ecosystems [1,2,3]. The pest primarily feeds on tender tissues of forage grasses such as leaf tips and shoot tips during its larval stage. More than 20 species of forage grasses, including species of the Cyperaceae, Poaceae, Fabaceae, Polygonaceae, and Rosaceae families, are consumed by its voracious larvae. This polyphagous and selective feeding behavior seriously affects the flowering of forage grasses and inhibits the growth and normal development of forage. Meanwhile, the growth of poisonous weeds gradually increases, leading to the degradation of grassland plant community structure and the deterioration of grassland ecological environments [4,5,6,7]. Additionally, larval exuviae (shed skin) and cocoons are poisonous to livestock, leading different types of mouth sores and broken tongue disease in domestic animals, and they are also poisonous to humans, causing serious skin irritations [8,9]. In recent years, due to outbreaks occurring within a relatively concentrated period and being difficult to control, as well as global climate change, populations of this pest have frequently exploded and caused disasters. It has become a major factor limiting the sustainable utilization and healthy development of alpine meadows.

For a long time, herders have mainly relied on spraying chemical pesticides to control caterpillars on grassland. However, the long-term, large-scale, and improper use of chemical pesticides not only causes problems such as environmental pollution and pesticide residues but also readily kills environmental non-target beneficial organisms. This leads to negative consequences on grasslands, including a decline in biodiversity and the phenomenon of ecosystem environmental disequilibrium, and is unfavorable for the healthy and sustainable development of grass and the animal industry [10,11]. With advancements in science and technology and the strengthening of ecological civilization, Qinghai Province is also striving to implement the strategy of “Give priority of ecological protection, promote high-quality development, and create high-quality life” and to make Qinghai Province an exporter of green and organic agricultural and livestock products. Evidently, chemical control far from meets the requirements for green prevention and control of insect pests, and using natural enemy insects and biocontrol microbial agents to control grassland caterpillars has become critical approach. To date, parasitoid wasps [12,13], parasitoid flies [14,15,16], entomopathogenic bacteria [17], insect pathogenic viruses [18], and entomopathogenic nematodes [19] have been explored as sustainable biological alternatives to biological control strategies for the management of grassland caterpillar.

As crucial microbial resources, entomopathogenic fungi (EPF) have attracted growing interest for the control of agricultural and forestry pests in recent decades as they represent the largest group of insect-pathogenic microorganisms. According to incomplete statistics, more than 100 genera and over 1000 species of EPF have been reported worldwide, and more than 750 species can cause natural epizootics in insect populations [20,21,22,23,24]. In addition, EPF also have other advantages such as being environmentally friendly, highly efficient, able to be mass-produced, safe for humans, and free from residual effects [25,26,27,28]. However, their application for controlling the grassland caterpillar has remained relatively limited. The selection of strains of entomopathogenic fungi in biological control programs depends not only on host specificity but also on geographical differences [29,30,31]. Practical studies have proven that the pathogens isolated from the original host generally provide more specific and effective control efficiency for target insects [32]. Therefore, isolating and utilizing biocontrol strains against local pests is the key technical link for implementing eco-friendly pest management. In this study, an entomopathogenic fungus strain was isolated from infected *G. qinghaiensis* larvae collected from an alpine rangeland in Gangcha County, Haibei Tibetan Autonomous Prefecture, Qinghai Province. The strain was identified based on morphological characteristics and ITS-rDNA sequences. Moreover, its larvicidal efficacy against grassland caterpillar under laboratory conditions and control efficacy on grasslands were comprehensively evaluated. This research aims to provide new candidate strain resources and theoretical basis for the green control of grassland pests.

## 2. Materials and Methods

### 2.1. Fungal Strain and Preparation

Entomopathogenic fungi: One naturally infected larva of *G. qinghaiensis* was collected from an alpine grassland in Gangcha County, Haibei Tibetan Autonomous Prefecture, Qinghai Province (37°23′35″ N, 100°30′40″ E and altitude of 3200 m), on 20 June 2023, and then maintained at 4 °C at the Key Laboratory of Agricultural Integrated Pest Management of Qinghai Province of Qinghai University. Sabouraud’s dextrose yeast extract agar (SDAY) medium (40 g dextrose, 10 g yeast extract, 10 g peptone, 20 g agar, 1000 mL distilled water) was employed for strain isolation [33].

Target insects: Healthy larvae of *G. qinghaiensis* were collected from an alpine grassland in Haiyan County, Haibei Tibetan Autonomous Prefecture, Qinghai Province, China (37°23′35″ N, 100°30′40″ E, altitude of 3200 m). They were fed on the above-ground organs of *Festuca sinensis* in a laboratory and reared in ventilated plastic boxes (17.0 cm × 11.5 cm × 7.0 cm). The larvae were transferred to a growth chamber (MGC-450HP, Shanghai One Instrument Science Instrument Co., Ltd., Shanghai, China) with 25 ± 1 °C temperature, 70 ± 5% relative humidity, and a 16 h light: 8 h dark photoperiod, and then the healthy, uniform-sized fourth-instar larvae were selected for experimental use.

Insecticides tested: Four insecticides, namely, abamectin, spinetoram, spinosad, and pyrethrin, were selected for experimental use. Specific information on the active drugs is shown in Table 1.

### 2.2. Isolation of Strain GC23620

A cadaver of a *G. qinghaiensis* larva was placed in a Petri dish lined with moist filter paper and incubated at room temperature for 2–3 days. Some white mycelia could be seen on the insect surface. Then, the fresh mycelium was transferred onto Petri dishes containing SDAY medium under aseptic conditions using the point-inoculation method. When the white mycelia re-emerged after cultivation, the cutting-edge hyphae picking method was used for purification. The entire isolation process was conducted under the conditions of 25 ± 1 °C temperature, 80 ± 5% relative humidity, and a 16 h light/8 h dark photoperiod. The isolated and purified strain was designated GC23620 and stored in a refrigerator at 4 °C.

### 2.3. Morphological Identification of Strain GC23620

The purified target strain was inoculated onto Petri dishes containing SDAY medium and incubated under the conditions of 25 ± 1 °C temperature, 80 ± 5% relative humidity, and a 16 h light/8 h dark photoperiod. Colony growth was regularly observed, and colony color was recorded. A small amount of grown mycelium was teased apart from the colony using an inoculating needle and mounted on a glass slide with a drop of sterile water. Morphological characteristics were observed under a light microscope. After sporulation, conidial morphology was microscopically examined, and the diameters of 20 randomly selected conidia were measured. Based on the characteristics of the colonies, mycelia, and conidia and by referring to the morphological features of identified *Beauveria* species summarized by Rehner et al. [34], the isolated strain was preliminarily identified using morphological examination.

### 2.4. Molecular Identification of Strain GC23620

Using a sterile pipette tip, a small amount of fungal biomass was scraped off and transferred into a sterile mortar. It was rapidly ground into a powder in liquid nitrogen and further transferred to a 1.5 mL centrifuge tube. DNA was extracted using a Solarbio Fungal Genomic DNA Extraction Kit (Solarbio Science & Technology Co., Ltd., Beijing, China) as per the manufacturer’s instructions. The extracted DNA was stored at −20 °C and subsequently used for PCR. Universal primers ITS1 (5′-TCCGTAGGTGAACCTGCGG-3′) and ITS4 (5′-TCCTCCGCTTATTGATATGC-3′), synthesized by Sangon Biotech (Shanghai) Co., Ltd., Shanghai, China, were used to amplify the rDNA-ITS region. The PCR reaction mixture (25 μL) consisted of 12.5 μL 2× Taq PCR Mix [TIANGEN Biotech (Beijing) Co., Ltd., Beijing, China], 1 μL ITS1, 1 μL ITS4, 1 μL template DNA, and 9.5 μL ddH_2_O. The PCR program was set as follows: predenaturation at 94 °C for 3 min; 30 cycles of denaturation at 94 °C for 30 s, annealing at 55 °C for 30 s, and extension at 72 °C for 1 min; final extension at 72 °C for 5 min; and holding at 4 °C. The PCR products were analyzed using 1.0% agarose gel electrophoresis. The samples were collected and sent for sequencing (Sangon Biotech (Shanghai) Co., Ltd., Shanghai, China).

Based on the sequencing results, low-quality bases at both ends of the obtained sequence were trimmed. The optimized sequence was further subjected to BLAST+ 2.16.0 (http://blast.ncbi.nlm.nih.gov/Blast.cgi, accessed on 11 November 2023) to identify the most similar representative strains. The corresponding sequences were downloaded. Phylogenetic trees were constructed in MEGA 11.0 using the neighbor-joining method (NJ) and the Kimura two-parameter (K2P) model. Bootstrap analysis with 1000 replicates was conducted to evaluate the stability of the tree topology.

### 2.5. Larvicidal Efficacy of Strain GC23620 Against G. qinghaiensis Larvae Under Laboratory Conditions

The strain GC23620 was inoculated onto the Petri dishes containing SDAY medium by using streak plate method and the fungal culture was maintained at 25 ± 1 °C temperature, 80 ± 5% relative humidity and a 16 h light: 8 h dark photoperiod for 7 days.

The conidia were gently scraped off with a sterile inoculating loop, placed in a conical flask containing 50 mL sterile water with 0.01% Tween·80 (*w*/*v*) (Beijing Solaibao Technology Co., Ltd., Beijing, China), and aggressively shaken for 10 min. When the conidia were evenly dispersed, the hyphae were separated with sterile gauze. The number of conidia per mL was counted using a Neubauer hemocytometer chamber (Shanghai Anxin Optical Instrument Manufacturing Co. Ltd., Shanghai, China). The experimental concentration was adjusted to 1.05 × 10^9^ conidia/mL, and then was diluted to 1.05 × 10^8^, 1.05 × 10^7^, 1.05 × 10^6^, and 1.05 × 10^5^ conidia/mL for subsequent experiments.

The fourth-instar larvae of *G. qinghaiensis* (15 larvae/replication—three replications) were immersed into conidial suspensions with the above five concentrations and sterile water containing 0.01% Tween·80 for 10 s [35,36]. The treated larvae were transferred into sterile plastic containers (D = 7 cm, H = 9 cm) and supplied with fresh *F. sinensis*. The stem bottoms of forage grasses were wrapped with water-moistened absorbent cotton to prevent wilting and changed once every 48 h. One larva was placed in each box. Every treatment was performed at 25 ± 1 °C temperature, 80 ± 5% relative humidity, and a 16 h light: 8 h dark photoperiod for 8 days in a growth chamber. Mortality was observed at regular intervals for 24 h. During inspection, larva was gently prodded with forceps; if the larval body was rigid and unable to curl up, it was considered dead. Dead larvae were placed in Petri dishes lined with moist filter paper and incubated at 25 ± 1 °C for 3–5 days. The mycelia or conidia on larval carcasses examined using a light microscope could further confirm that the death was caused by mycosis. The cumulative corrected mortality [37] was calculated as follows:

Cumulative corrected mortality (%) = (mortality observed − mortality in control)/(100 − mortality in control) × 100%.(1)

Furthermore, the leaf dipping method [38,39] was used to determine the virulence of strain GC23620 against *G. qinghaiensis* larvae under the same concentrations of conidial suspension. Fresh forage segments were prepared as described above and immersed into conidial suspensions and sterile water containing 0.01% Tween·80 for 10 s. The treated forage segments were transferred into sterile plastic containers (D = 7 cm, H = 9 cm), and one fourth-instar larva was placed in each container. Each treatment included three replicates, with 15 larvae per replicate. Larvae were reared and observed under the same conditions as described above, and the assessment procedures were the same as those for the insect immersion method.

### 2.6. Control Efficacy of Strain GC23620 Against G. qinghaiensis Larvae on Grassland

Based on the virulence data obtained under laboratory conditions, a relatively low concentration of 1.05 × 10^5^ conidia/mL was selected. The conidial suspension of strain GC23620 was prepared with liquid culture medium of SDAY containing 0.01% Tween·80, and then the suspension was shaken at room temperature with speed of 165 rpm/min in a constant-temperature shaker oscillator for 48 h. The fermentation solution was intended for experimental use and the CK control was sterile water containing 0.01% Tween·80. In addition, abamectin, spinetoram, spinosad, and pyrethrin were selected as insecticides for the control experiment. The active drugs of the above four insecticides were first dissolved in acetone for mother liquor preparation with the concentration of 10.00%, and then diluted with sterile water containing 0.01% Tween·80 (*w*/*v*) into LD_50_.

The experimental area was selected in the alpine meadow of Laorigen Village, Mole Town, Qilian County, Haibei Prefecture, Qinghai Province (37°43′26″ N, 100°38′53″ E, altitude 3470.34 m). Each treatment plot was approximately 200.0 m^2^ (20.0 m × 10.0 m), with three replicates per treatment. Plots were separated by 1.0 m and arranged in a randomized design. The initial population density of *G. qinghaiensis* larvae in each treatment plot was surveyed before spraying using a checker-board five-point method (with each point having an area of 1.0 m^2^). Spraying was conducted on 15 July 2025, which was a cloudy day without rainfall. Each treated solution, including four insecticides, the fermentation of *B. bassiana* GC23620, and the control, was sprayed evenly within its respective experimental plot using a backpack electric sprayer (Model: SX-MD161). Post-treatment, the number of surviving larvae was recorded at 3, 7, 15, and 21 days in each plot. Sampling was conducted as described above. The population reduction rate and the corrected control efficacy [40] were calculated as follows:

Population reduction rate (%) = [(pretreatment population density − post-treatment population density)/pretreatment population density] × 100%.(2)

Field control efficacy (%) = [(population reduction rate in treatment − population reduction rate in control)/(1 − population reduction rate in control)] × 100%.(3)

### 2.7. Data Analysis

Experimental data were processed and calculated using Microsoft Office 2019 and expressed as mean ± standard error (SE). All data were analyzed using one-way ANOVA, and the significant differences (at the 5% and 1% levels of significance) were analyzed using Duncan’s new multiple-range test. All data were analyzed using the Statistical Product Service Solutions (SPSS) software, version 20.0.

Bioassay data were analyzed using the time–dose–mortality (TDM) model to determine the interactions between strain GC23620 and *G. qinghaiensis* larvae [41,42], and the lethal dose (LD_50_ and LD_90_) and lethal time (LT_50_ and LT_90_) were both obtained through model simulation. All data were analyzed using the Data Processing System (DPS) software, version 14.0 [43].

## 3. Results

### 3.1. Taxonomic Identification of Strain GC23620

#### 3.1.1. Morphological Identification

**Figure 1:**
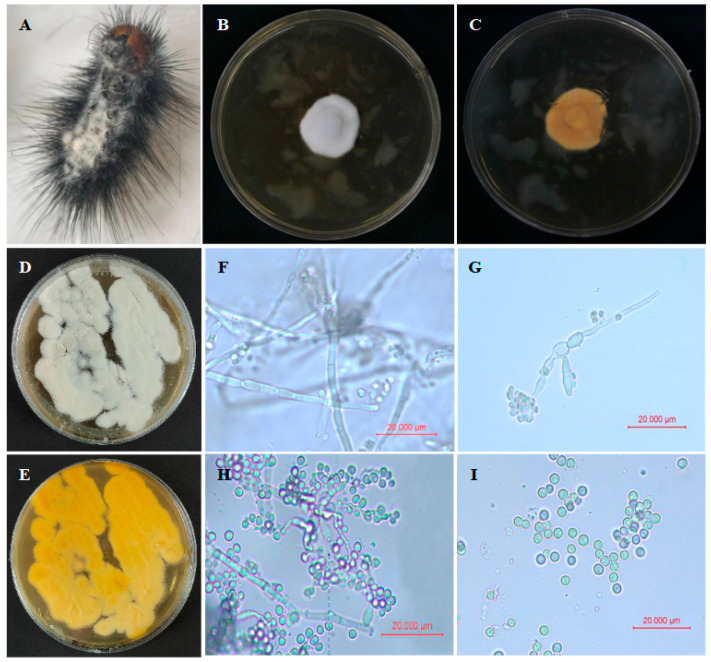
Infected *G. qinghaiensis* larva and morphological characteristics of strain GC23620. (**A**): Symptoms of infected *G. qinghaiensis* larva. (**B**,**C**): Culture produced via the point inoculation method on the front and back sides of SDAY medium (on the 8th day). (**D**,**E**): Culture produced via the plate streaking method on the front and back sides of SDAY medium (on the 8th day). (**F**): Mycelial morphology. (**G**,**H**): Conidiogenous structure morphology in early and later periods. (**I**): Conidial morphology.

The dead *G. qinghaiensis* larvae infected with strain GC23620 collected from the grassland showed typical characteristics, as shown in Figure 1A. For the strain GC23620 isolated from infected larvae on SDAY medium, colonies were initially white and short-velvety. After 3 days, the colonies became flat, white, and floccose, with regular margin and radial growth spreading outward. Sporulation began after 6 days. At this time, the colony center was slightly raised; conidial masses formed a ring-like accumulation that expanded outward, surrounded by scattered mycelia. On the reverse side, the colony center appeared dark yellowish-brown and opaque, with the color gradually becoming lighter from the central spot toward the periphery (Figure 1B–E).

Light microscopic examination revealed that strain GC23620 possessed hyphae that were branched and septate, with a smooth and colorless surface, and with a width of 2.45 ± 0.05 μm (Figure 1F). Conidiophores arose from vegetative hyphae and produced conidia in a sympodial manner at their apices, forming spike-like conidiogenous structures (Figure 1G,H). Conidia were spherical or subspherical, transparent, and smooth, with an average diameter of approximately 3.43 ± 0.18 μm (Figure 1I). Based on the morphological characteristics of the colony, hyphae, conidiogenous structures, and conidia as described previously [44], the characteristics of strain GC23620 were consistent with the those of the genus *Beauveria*.

#### 3.1.2. Molecular Identification

**Figure 2:**
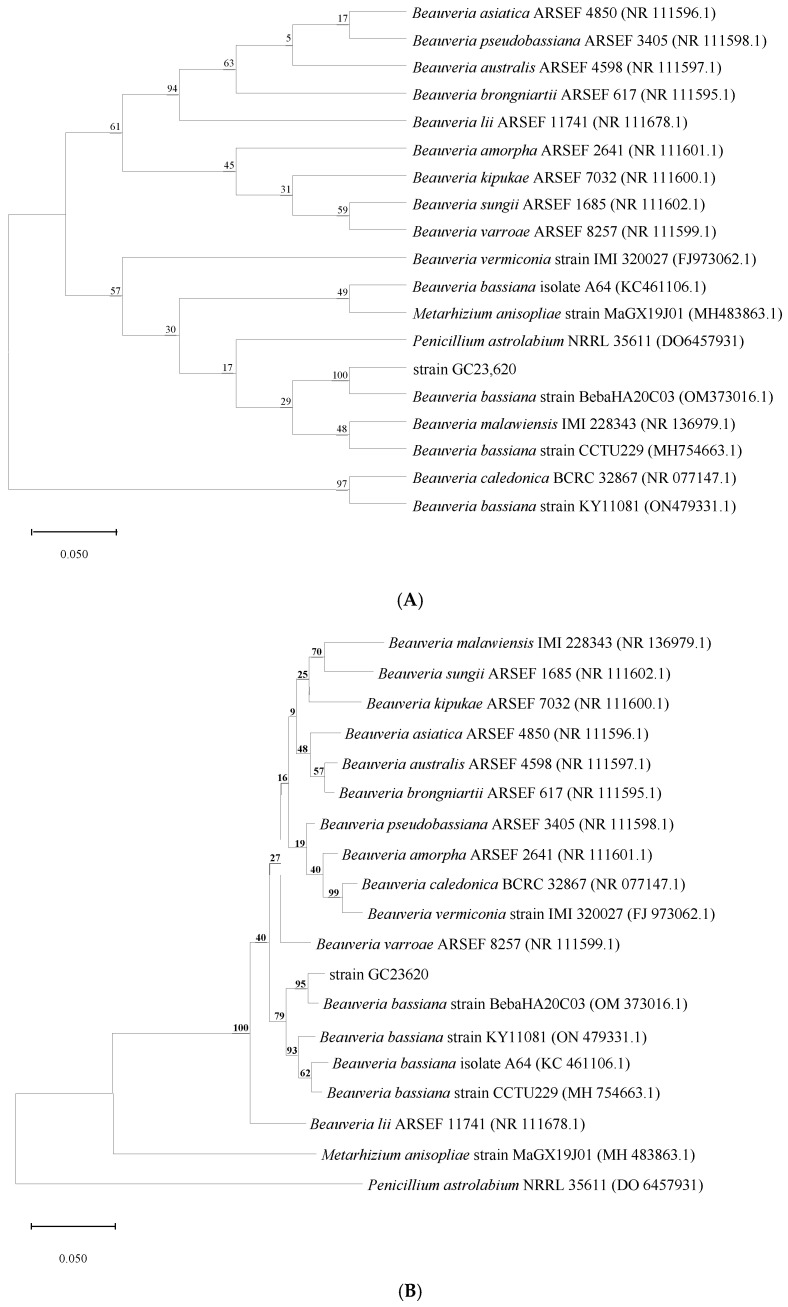
Phylogenetic tree of the isolated strain GC23620 and other close related strains. The numbers in parentheses represent GenBank accession numbers. The numbers in each branch point denote the percentages supported by bootstrap. The scale bar of the branch is 0.05. (**A**): Based on the neighbor-joining method model; (**B**): based on the Kimura two-parameter model.

The rDNA-ITS sequence fragment of the isolated strain was amplified using PCR. Sequencing results revealed that the amplified fragment was 545 bp in length. After removing low-quality sequences from both ends, a BLAST homology comparison was performed in the GENBANK database. The analysis revealed that strain GC23620 clustered with *B. bassiana* in the database with high bootstrap support. Relevant reference sequences were selected, and a phylogenetic tree was constructed using MEGA 11 (Figure 2). Strain GC23620 shares 100% homology with B. bassiana strain BebaHA20C03 (OM373016.1) according to the NJ model and shares 95% homology based on the K2P model. Based on the combined evidence from morphological characteristics and ITS sequence similarity analysis, the isolate was ultimately identified as *B. bassiana* and designated GC23620.

### 3.2. Larvicidal Efficacy of B. bassiana GC23620 Against G. qinghaiensis Larvae Under Laboratory Conditions

#### 3.2.1. Symptoms of Infection of *G. qinghaiensis* Larvae by *B. bassiana* GC23620

**Figure 3:**
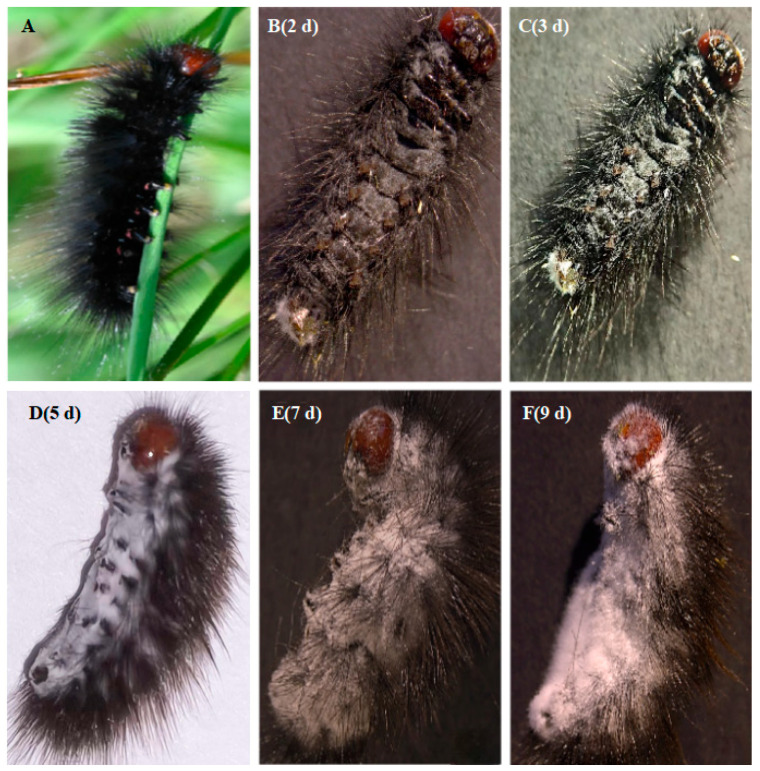
Symptoms of *B. bassiana* GC23620 infection in larvae of *G. qinghaiensis*. (**A**): Healthy larvae; (**B**–**F**): Symptoms of infection of *G. qinghaiensis* larvae by *B. bassiana* GC23620 from the 2nd day to the 9th day.

After infection by *B. bassiana* GC23620, *G. qinghaiensis* larvae exhibited a reduction in eating and sluggish movement, resulting in the larval body becoming rigid and a decrease in luster, and then showed mycoses symptoms. White ‘star-like’ mycelia grew from the intersegmental membranes of the larval abdomens starting on the second or third day after infection. After another 1–2 days, white floccose mycelia increased on the larval surface and the body was encased entirely in mycelium. A small number of white conidia appeared on the larvae on the fifth or sixth day post-infection. As the conidia matured and accumulated, forming conidial masses, they ultimately enveloped the entire larval body on the seventh or eighth day after infection (Figure 3). The conidia from the surface of infected larvae were examined using a microscope, and it was revealed that their morphology was consistent with that of *B. bassiana* GC23620.

#### 3.2.2. Cumulative Corrected Mortality of *G. qinghaiensis* Larvae at Various Time Intervals

**Figure 4:**
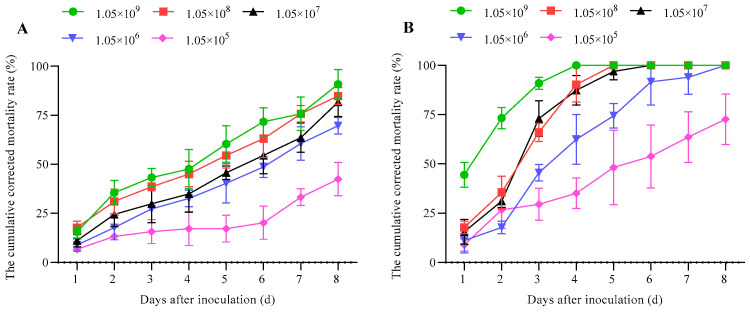
The cumulative corrected mortality rate of *G. qinghaiensis* larvae inoculated with *B. bassiana* GC23620 using five conidia concentrations and two different methods. Data are presented as mean ± SD. (**A**): Insect immersion method; (**B**): leaf dipping method.

The mortality of fourth-instar larvae of *G. qinghaiensis* observed in two different immersion bioassays was concentration- and time-dependent. One day after inoculation with *B. bassiana* GC23620, the death of larvae began to be observed. Furthermore, cumulative corrected mortality varied in response to days. With an inoculation concentration of 1.05 × 10^9^ conidia/mL using the insect immersion method, the mortality was 90.91 ± 7.42% at 8 days after inoculation (DAI), and the mortality reached 84.85 ± 4.29%, 81.82 ± 7.42%, 69.70 ± 4.29%, and 42.42 ± 8.57%, respectively, during the same post-inoculation period with inoculation concentrations of 1.05 × 10^8^, 1.05 × 10^7^, 1.05 × 10^6^, and 1.05 × 10^5^ conidia/mL (Figure 4A). Comparatively, the mortality reached 100.00 ± 0.00% at 4 DAI using the leaf dipping method with an inoculation concentration of 1.05 × 10^9^ conidia/mL, at 5 DAI with 1.05 × 10^8^ conidia/mL, at 6 DAI with 1.05 × 10^7^ conidia/mL, and at 8 DAI with 1.05 × 10^6^ conidia/mL. The mortality reached 72.73 ± 12.86% at 8 DAI with an inoculation concentration of 1.05 × 10^5^ conidia/mL (Figure 4B). In summary, the conidia of *B. bassiana* GC23620 showed higher pathogenicity in the four-instar larvae of *G. qinghaiensis* when using both the insect immersion and leaf dipping methods.

#### 3.2.3. TDM Model of *B. bassiana* GC23620 Against *G. qinghaiensis* Larvae

The observed responses of *G. qinghaiensis* larvae fit the TDM model, with an acceptable homogeneity fit based on the Hosmer–Lemeshow statistic (*p* ≥ 0.05) (Table 2) for both the insect immersion method (*χ^2^* = 1.08, *df* = 8) and the leaf dipping method (*χ^2^* = 5.15, *df* = 7). In addition, the *t*-test statistics for all parameters estimated also reached a highly significant level (*p* < 0.01), indicating that the SEs were extremely small relative to the parameter estimates. This demonstrated that both the dose effect and the time effect of the tested strain were highly significant.

The estimated parameters (*β*) for the concentration effect of strain GC23620 were 0.30 and 0.56 for the insect immersion and leaf dipping methods, indicating that *B. bassiana* GC23620 was more pathogenic to larvae when applied using the leaf dipping method. The parameter for the conditional time effect (*γ_i_*) reached the highest value at 8 DAI, indicating that the highest mortality was on the eighth day.

#### 3.2.4. Dose–Response Effects of *B. bassiana* GC23620 Against *G. qinghaiensis* Larvae Infection

**Figure 5:**
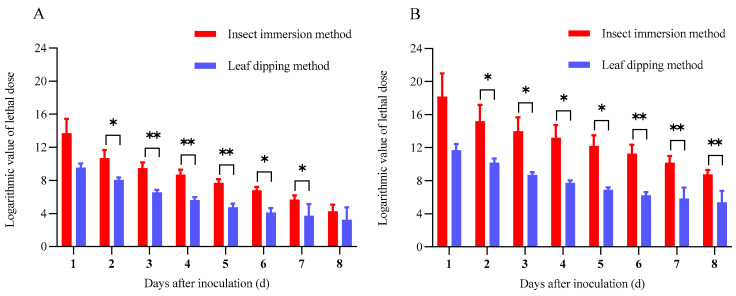
Logarithmic value of lethal dose of *B. bassiana* GC23620 against *G. qinghaiensis* larvae inoculated by different methods. Data are presented as mean ± SD. * *p* < 0.05; ** *p* < 0.01. (**A**): Logarithmic value of median lethal dose (LD_50_) (at which 50% mortality was observed). (**B**): Logarithmic value of median lethal dose (LD_90_) (at which 90% mortality was observed).

The dose effect of *B. bassiana* GC23620 infection on *G. qinghaiensis* larvae at different time intervals was estimated using the TDM model. The lethal doses (LD_50_ and LD_90_) in the two inoculation methods were all gradually decreased as the number of inoculation days increased (Figure 5). The logarithm of LD_50_ for the insect immersion method decreased from 13.70 ± 1.74 at 1 DAI to 4.27 ± 0.82 at 8 DAI, meaning that LD_50_ declined from 5.02 × 10^13^ to 1.85 × 10^4^ conidia/mL. The logarithm of LD_90_ for the same method decreased from 18.19 ± 2.79 to 8.76 ± 0.55, corresponding to a decline from 1.56 × 10^18^ to 5.76 × 10^8^ conidia/mL. By comparison, the logarithms of LD_50_ and LD_90_ for the leaf dipping method decreased from 9.55 ± 0.51 and 11.68 ± 0.77 to 3.24 ± 1.51 and 5.37 ± 1.40, respectively; that is, LD_50_ declined from 3.51 × 10^9^ to 1.74 × 10^3^ conidia/mL, and LD_90_ declined from 4.79 × 10^11^ to 2.37 × 10^5^ conidia/mL.

In terms of the significance levels of LD_50_ with the two inoculation methods, there was no significance at 1 DAI and 8 DAI (1 DAI: *F*_1,4_ = 7.16, *p* = 0.06; 8 DAI: *F*_1,4_ = 1.80, *p* = 0.25). The LD_50_ values for the leaf dipping method at the infection periods of 2 DAI, 6 DAI, and 7 DAI were lower than those for the insect immersion method (2 DAI: *F*_1,4_ = 8.86, *p* = 0.04; 6 DAI: *F*_1,4_ = 17.41, *p* = 0.01; 7 DAI: *F*_1,4_ = 11.58, *p* = 0.03), and the LD_50_ values for the leaf dipping method at the infection periods of 3 DAI, 4 DAI, and 5 DAI were significantly lower than those for the insect immersion method (3 DAI: *F*_1,4_ = 29.64, *p* < 0.01; 4 DAI: *F*_1,4_ = 28.20, *p* < 0.01; 5 DAI: *F*_1,4_ = 24.79, *p* < 0.01). Comparatively, there was no difference in the LD_90_ at 1 DAI with only these two inoculation methods (*F*_1,4_ = 6.91, *p* = 0.06). The LD_90_ for the leaf dipping method was lower than that for the insect immersion method from 2 DAI to 5 DAI (2 DAI: *F*_1,4_ = 8.01, *p* = 0.05; 3 DAI: *F*_1,4_ = 13.69, *p* = 0.02; 4 DAI: *F*_1,4_ = 16.95, *p* = 0.01; 5 DAI: *F*_1,4_ = 18.42, *p* = 0.01), and the LD_90_ for the leaf dipping method was significantly lower than that for the insect immersion method from 6 DAI to 8 DAI (6 DAI: *F*_1,4_ = 21.68, *p* < 0.01; 7 DAI: *F*_1,4_ = 25.54, *p* < 0.01; 8 DAI: *F*_1,4_ = 71.62, *p* < 0.01).

#### 3.2.5. Time Effect of *G. qinghaiensis* Larvae Infection by *B. bassiana* GC23620

The time effects of *B. bassiana* GC23620 infection on *G. qinghaiensis* larvae were estimated by interpolation in the fitted TDM model, which indicated that the lethal times (LT_50_ and LT_90_) using the two inoculation methods were reduced after increasing the conidial concentration (Table 3). There were no differences in either LT_50_ or LT_90_ under the insect immersion method with inoculation concentrations from 10^6^ conidia/mL to 10^8^ conidia/mL (LT_50_: *F*_2,6_ = 2.82, *p* = 0.14; LT_90_: *F*_2,6_ = 2.61, *p* = 0.15), and the lowest LT_50_ and LT_90_ at a concentration of 10^8^ conidia/mL were 3.16 d and 11.24 d, respectively. Comparatively, there was no difference in the LD_90_ under the leaf dipping method with inoculation concentrations from 10^6^ conidia/mL to 10^8^ conidia/mL (*F*_2,6_ = 4.19, *p* = 0.07), but there was a significant difference in LD_50_ at the same inoculation concentration (*F*_2,6_ = 7.02, *p* = 0.03). The lowest LT_50_ and LT_90_ at a concentration of 10^8^ conidia/mL under the leaf dipping method were 1.75 d and 2.52 d, respectively. When the inoculation concentration was determined, the values of LT_50_ for the leaf dipping method were all significantly lower than those under the insect immersion method (10^6^ conidia/mL: *F*_1,4_ = 19.00, *p* = 0.01; 10^7^ conidia/mL: *F*_1,4_ = 18.65, *p* = 0.01; 10^8^ conidia/mL: *F*_1,4_ = 26.61, *p* < 0.01), and the LT_90_ presented the same trend (10^6^ conidia/mL: *F*_1,4_ = 71.22, *p* < 0.01; 10^7^ conidia/mL: *F*_1,4_ = 41.42, *p* < 0.01; 10^8^ conidia/mL: *F*_1,4_ = 94.10, *p* < 0.01).

### 3.3. Control Efficacy of B. bassiana GC23620 Against G. qinghaiensis Larvae on Grassland

The average initial population density of *G. qinghaiensis* larvae in the treatment plot was 203.33 ± 16.50 individuals/m^2^ before spraying *B. bassiana* GC23620 fermentation, and the corrected control efficacy reached 33.18 ± 4.19% at 3 days post-treatment. By comparison, the average initial population densities of *G. qinghaiensis* larvae in the other plots were 70.33 ± 7.41 individuals/m^2^ (spinetoram), 52.00 ± 10.61 individuals/m^2^ (pyrethroids), 82.00 ± 2.45 individuals/m^2^ (abamectin), 51.33 ± 12.66 individuals/m^2^ (spinosad), and 112.67 ± 19.34 individuals/m^2^ (CK). At 3 days post-treatment, the corrected control efficacies of the above four treatments were 22.44 ± 7.60%, 13.53 ± 16.86%, 28.47 ± 2.71% and 13.44 ± 23.76% respectively. There were no significant differences (*F*_4,10_ = 0.84, *p* = 0.53) in control efficacy among the five treatments at 3 days post-treatment. With the extension of time after spraying, the control efficacy increased after above five treatments. Nevertheless, there were also no significant differences (*F*_4,10_ = 1.20, *p* = 0.37) in control efficacy among the five treatments at 21 days post-treatment. The control efficacy reached 84.27 ± 3.32% after spraying *B. bassiana* GC23620 fermentation and 74.95 ± 7.05% after spraying abamectin at the same time point. Comparatively, spraying spinosad showed the lowest control efficacy of 59.86 ± 17.45% (Table 4). It was indicated that the application of *B. bassiana* GC23620 fermentation at a concentration of 1.05 × 10^5^ conidia/mL via spraying could provide a certain level of control against *G. qinghaiensis* in grassland.

## 4. Discussion

In this study, an EPF strain was isolated from the surface of naturally infected *G. qinghaiensis* larvae. Based on morphological characteristics and molecular phylogenetic analysis, this strain was identified as *B. bassiana*, with the designation GC23620. The EPF *B. bassiana* play a crucial role as a biological control agent against agricultural and forestry pests, owing to their wide host range of more than 750 insect species across 149 families in 15 orders [45,46]. In China, *B. bassiana* has been used successfully as a biocontrol resource to control *Helicoverpa armigera* [47], *Plutella xylostella* [48], *Hyphantria cunea* [49], *Spodoptera exigua* (Hübner) [50], *Ostrinia furnacalis* [33], *Spodoptera frugiperda* [51,52], *Spodoptera litura* [53], *Dendrolimus punctatus* [54], locusts [55,56], weevil pests [57,58,59,60,61,62], *Frankliniella occidentalis* [63], *Leptinotarsa decemlineata* [64], *Monochamus alternatus* [65], *Diaphorina citri* [66], *Bemisia tabaci* [67], *Delia antiqua* [68], and the red fire ant *Solenopsis invicta* [69]. However, there is currently a significant shortage of highly virulent biocontrol strains effective against grassland caterpillar. The pathogenicity of *B. bassiana* GC23620 was tested against fourth-instar larvae of *G. qinghaiensis* using the insect immersion method under laboratory conditions, and the data revealed dose-dependent mortality. As the concentration of spores increased, the mortality rate of larvae increased significantly and exhibited a total mortality rate of 90.91 ± 7.42% at a concentration of 1.05 × 10^9^ conidia/mL at 8 DAI. The lowest LD_50_ of 1.85 × 10^4^ conidia/mL was obtained at 8 DAI. The LT_50_ was 4.51 days at an inoculation concentration of 1.05 × 10^6^ conidia/mL. The results showed that the strain of *B. bassiana* GC23620 has potential for the green control of grassland caterpillar.

However, the larvicidal efficacy of EPF against target pests was also influenced by different application methods independently of the genetic diversity and population heterogeneity of different strains. Similar findings on the toxicity of *Metarhizium flavoviride* strain Ma130821 were observed in *Holotrichia parallela* Motschulsk larvae when treated via a potted soil method, circling fertilization, and hole application. The use of the potted soil method could improve uniformity, thereby increasing the contact opportunities between grubs and conidia power. Ultimately, the accumulative mortality was 95.69% using the potted soil method, and the effect was superior to the other two methods [70]. Zheng et al. [71] inoculated *Phthorimaea operculella* pupae with conidia of *Cordyceps tenuipes* by using an immersion method and soil treatment, respectively. The results showed that drenching the soil surface with conidial suspensions was the most effective method for field application with short lethal time of 2.6 days and low lethal concentrations of 1.10 × 10^5^ conidia/g. Fu et al. [72] also reported that the use of the soil mixing method for *Beauveria bassiana* (Bals.) Vuill conidia resulted in better pathogenicity to *Myllocerinus aurolineatus* larvae with LC_50_ of 1.11 × 10^3^ conidia/mL and LT_50_ of 7.84 days compared with the immersion method. Our study reported that *B. bassiana* GC23620 exhibited markedly higher virulence against *G. qinghaiensis* larvae when using the leaf dipping method compared to the insect immersion method, with the lowest LD_50_ of 1.74 × 10^3^ conidia/mL at 8 DAI. When the inoculation concentration was 1.05 × 10^6^ conidia/mL, the cumulative corrected mortality and LT_50_ were 100.00% and 2.40 days respectively. This may be because the conidia adhered to surface regions of the insect’s body such as epidermal folds, depressions, and body joints when using the method of insect immersion for inoculation. During the infection process, fungi produce specialized infection structures, such as penetration pegs and appressoria, which allow growing hyphae to break through the host’s integument with the aid of extracellular hydrolyzing enzymes and mechanical stress. If either of these two stages is hindered, further infection cannot proceed [73,74,75]. Comparatively, when treated with the leaf dipping method, the conidia first attached to surface regions of the plants, and then the plants were all eaten by the insects, thus entering their gut. Once inside the host, fungal biomass continuously increases under favorable internal conditions, enabling the production of toxins and metabolites and providing a nutritional basis for the secretion of virulence-related compounds that contribute to host death [76]. Additionally, conidia on the plants could also reattach to the surface of the insect through the insects feeding on the plants, and then invade the cuticle of the insect through a combination of mechanical pressure and enzymatic action. The infection efficiency was increased under these two infection processes.

Field experiments are an important approach for evaluating the biocontrol potential of EPF against target pests. Some EPF exhibit strong pathogenicity under laboratory conditions, but their performance under field conditions may be limited by numerous factors. The speed of conidial germination and conidial viability are critical parameters associated with virulence. Notably, conidial germination is an indispensable process for initiating infection by EPF [77,78]. Qinghai Province has a typical climate characteristic of the Qinghai–Tibet Plateau, such as long hours of hot sunshine, strong ultraviolet radiation, low humidity, and large temperature differences between day and night, and these conditions could cause slow growth of mycelium for conidia with low germination. To improve the control effect of *B. bassiana* GC23620 under field conditions, a fermentation solution that had been cultured for two days at room temperature conditions was used in this study in order that the conidia could fully germinate. Then, the fermentation solutions were sprayed on grasslands to control *G. qinghaiensis* larvae. The corrected control efficacy was only 33.18% at 3 days post-treatment, but the efficacy duration was 21 days post-treatment, with a corrected control efficacy of 84.27%. This result may have been because we did not take protective measures when spraying the fermentation solutions. Researchers have discovered that when releasing EPF manually in the field, the quantity of colony forming units (CFU) drops sharply regardless of whether a submerged or inoculated release method is used. The quantity of CFU first decreased rapidly in the early stage [79,80]. Subsequently, the decline has been slowing and eventually it may fluctuate in a certain range or increased slightly [81]. The reason for this requires further study. To address the limitations of the *B. bassiana* strain GC23620 in biological control, the synergistic effects of *B. bassiana* GC23620’s compounds and selected insecticides will be the focus of future studies, in order to provide technological support for reducing pesticide use and enhancing its effectiveness in controlling grassland pests.

## 5. Conclusions

In this study, an EPF strain was isolated from an infected larva of *G. qinghaiensis* and identified as *B. bassiana* based on morphological characteristics and ITS-rDNA sequence analysis and designated GC23620. The larvicidal efficacy of *B. bassiana* GC23620 conidia against *G. qinghaiensis* was evaluated by two methods of insect immersion and leaf dipping under laboratory conditions. In terms of lethal effects (larval mortality and lethal time), these two inoculation methods all exhibited good insecticidal performance. Moreover, the control efficacy on grassland remained as high as 84.27 ± 3.32% at 21 days post-treatment. This study provides a microbial resource for the development of biological control agents for grassland pests on alpine meadows.

## Figures and Tables

**Table 1:** Specific information on tested insecticides.

Insecticide	Active Ingredient (%)	Trademark Number	Manufacturer	Lethal Dose (ppm)
Abamectin	97.00%	A913373	Shanghai Macklin Biochemical Technology Co., Ltd., Shanghai, China	16.64
Pyrethrin	50.00%	P131624		23.13
Spinetoram	88.28%	B75139	Shanghai ZZBIO Co., Ltd., Shanghai, China	9.20
Spinosad	90.00%	B65604	Shanghai Yuanye Bio-Technology Co., Ltd., Shanghai, China	18.01

**Table 2:** Parameters of *B. bassiana* GC23620’s virulence against *G. qinghaiensis* larvae using different inoculation methods and estimated with TDM model.

Inoculation Methods	Conditional Mortality Model					Cumulative Mortality Model			
	Parameter ^a^	Estimated Value	Standard Error	*t*-Value Test ^b^	*p* Value	Parameter ^a^	Estimated Value	Var (τ_i_)	Cov (β, τ_i_)
Insect immersion method	*β*	0.30	0.11	2.86	0.0075	*β*	0.30	0.00	0.00
	*γ*_1_	−4.25	0.86	4.94	0.0001	*τ*_1_	−4.25	0.07	−0.01
	*γ*_2_	−4.05	0.86	4.74	0.0001	*τ*_2_	−3.46	0.06	−0.01
	*γ*_3_	−4.57	0.90	5.06	0.0001	*τ*_3_	−3.17	0.06	−0.01
	*γ*_4_	−4.82	0.95	5.07	0.0001	*τ*_4_	−3.00	0.06	−0.01
	*γ*_5_	−4.14	0.91	4.58	0.0001	*τ*_5_	−2.72	0.06	−0.01
	*γ*_6_	−3.96	0.90	4.41	0.0001	*τ*_6_	−2.47	0.06	−0.01
	*γ*_7_	−3.48	0.84	4.14	0.0002	*τ*_7_	−2.16	0.06	−0.01
	*γ*_8_	−2.96	0.84	3.54	0.0013	*τ*_8_	−1.79	0.05	−0.01
Hosmer−Lemeshow ^c^	*χ^2^* = 1.08, *df* = 8, *p* = 1.00								
Leaf dipping method	*β*	0.56	0.11	5.23	0.0001	*β*	0.56	0.00	0.00
	*γ*_1_	−5.69	0.88	6.45	0.0001	*τ*_1_	−5.69	0.26	−0.03
	*γ*_2_	−5.46	0.86	6.35	0.0001	*τ*_2_	−4.88	0.24	−0.03
	*γ*_3_	−4.62	0.82	5.61	0.0001	*τ*_3_	−4.05	0.22	−0.03
	*γ*_4_	−4.39	0.80	5.47	0.0001	*τ*_4_	−3.51	0.21	−0.03
	*γ*_5_	−4.04	0.77	5.23	0.0001	*τ*_5_	−3.05	0.19	−0.03
	*γ*_6_	−3.96	0.85	4.66	0.0001	*τ*_6_	−2.71	0.17	−0.02
	*γ*_7_	−4.32	1.02	4.23	0.0003	*τ*_7_	−2.53	0.16	−0.02
	*γ*_8_	−3.68	0.98	3.74	0.0011	*τ*_8_	−2.25	0.16	−0.02
Hosmer−Lemeshow ^c^	*χ^2^* = 5.15, *df* = 7, *p* = 0.64								

^a^ The subscripts γ and τ represent the specific day after inoculation. ^b^ The *t*-statistics for all the parameter estimates were highly significant (*p* < 0.0001). ^c^ Homogeneity hypothesis for the goodness of fit was accepted when *p* ≥ 0.05 in the Hosmer–Lemeshow test.

**Table 3:** Lethal time values (LT_50_ and LT_90_) for *G. qinghaiensis* larvae infected by *B. bassiana* GC23620 under the two different inoculation methods.

Lethal Time	Inoculation Methods	Inoculation Concentration (Conidia/mL)			
		1.05 × 10^6^	1.05 × 10^7^	1.05 × 10^8^	
LT_50_	Insect immersion	4.51 ± 0.63 aA	3.95 ± 0.68 aA	3.16 ± 0.35 aA	*F*_2,6_ = 2.82, *p* = 0.14
	Leaf dipping	2.40 ± 0.27 bA	1.87 ± 0.06 bB	1.75 ± 0.15 bB	*F*_2,6_ = 7.02, *p* = 0.03
		*F*_1,4_ = 19.00, *p* = 0.01	*F*_1,4_ = 18.65, *p* = 0.01	*F*_1,4_ = 26.61, *p* < 0.01	
LT_90_	Insect immersion	15.43 ± 1.78 aA	13.25 ± 2.31 aA	11.24 ± 1.25 aA	*F*_2,6_ = 2.61, *p* = 0.15
	Leaf dipping	3.80 ± 0.80 bA	2.72 ± 0.08 bAB	2.52 ± 0.21 bB	*F*_2,6_ = 4.19, *p* = 0.07
		*F*_1,4_ = 71.22, *p* < 0.01	*F*_1,4_ = 41.42, *p* < 0.01	*F*_1,4_ = 94.10, *p* < 0.01	

Data are presented as mean ± SD. Data in the same column followed by different lowercase letters represent significant differences at *p* < 0.05 with the same inoculation concentrations under different inoculation methods. Data in the same line followed by different uppercase letters indicate significant differences at *p* < 0.05 with the same inoculation method under different inoculation concentrations.

**Table 4:** Field efficacy of *B. bassiana* GC23620 against *G. qinghaiensis* larvae.

Treatment	Population Quantity	3 d Post-Treatment		7 d Post-Treatment		15 d Post-Treatment		21 d Post-Treatment	
		Population Decline Rate (%)	Corrected Control Efficacy (%)	Population Decline Rate (%)	Corrected Control Efficacy (%)	Population Decline Rate (%)	Corrected Control Efficacy (%)	Population Decline Rate (%)	Corrected Control Efficacy (%)
GC23620	203.33 ± 16.50	41.64 ± 12.10 aA	33.18 ± 4.19 aA	55.86 ± 2.18 aA	42.24 ± 6.25 aA	84.42 ± 3.14 aA	76.05 ± 3.24 aA	87.37 ± 2.76 aA	84.27 ± 3.32 aA
Spinetoram	70.33 ± 7.41	33.45 ± 7.74 abA	22.44 ± 7.59 aA	57.07 ± 6.73 aA	44.79 ± 0.80 aA	75.34 ± 1.09 abA	61.20 ± 6.81 abA	79.35 ± 3.69 aA	72.65 ± 11.45 aA
Pyrethrin	52.00 ± 10.61	27.18 ± 3.16 abA	13.53 ± 16.86 aA	51.32 ± 12.36 aA	33.36 ± 27.14 aA	63.68 ± 9.38 bA	42.20 ± 19.65 bA	75.85 ± 6.20 aA	68.09 ± 13.16 aA
Abamectin	82.00 ± 2.45	37.90 ± 10.49 aA	28.47 ± 2.71 aA	53.62 ± 1.39 aA	39.12 ± 7.88 aA	74.38 ± 2.59 abA	60.26 ± 3.83 abA	80.53 ± 2.27 aA	74.95 ± 7.05 aA
Spinosad	51.33 ± 12.66	28.08 ± 8.64 abA	13.44 ± 23.76 aA	49.70 ± 9.12 aA	33.24 ± 16.58 aA	68.39 ± 9.17 bA	51.88 ± 9.85 abA	67.33 ± 16.18 aA	59.86 ± 17.45 aA
Control group	112.67 ± 19.34	13.32 ± 13.11 bA	-	22.36 ± 11.30 bB	-	35.01 ± 8.14 cB	-	17.57 ± 17.08 bB	-
		*F*_5,12_ = 2.09, *p* = 0.14	*F*_4,10_ = 0.84, *p* = 0.53	*F*_5,12_ = 4.89, *p* = 0.01	*F*_4,10_ = 0.24, *p* = 0.91	*F*_5,12_ = 13.71, *p* < 0.01	*F*_4,10_ = 2.83, *p* = 0.08	*F*_5,12_ = 12.67, *p* < 0.01	*F*_4,10_ = 1.20, *p* = 0.37

Data are presented as mean ± SD. Data in the same column followed by different lowercase letters represent significant differences at *p* < 0.05 with different treatments, and different uppercase letters indicate significant differences at *p* < 0.01 with different treatments.

## Data Availability

Data are contained within the article.

## Abbreviations

The following abbreviations are used in this manuscript:

ANOVA	Analysis of variance
*B. bassiana*	*Beauveria bassiana*
DAI	Day after inoculation
DNA	Deoxyribonucleic acid
DPS	Data Processing System
EPF	Entomopathogenic fungi
CFU	Colony forming unit
*G. qinghaiensis*	*Gynaephora qinghaiensis*
LD	Lethal dose
LT	Lethal time
SDAY	Sabouraud dextrose agar medium supplemented with yeast extract
SD	Standard deviation
TDM	Time–dose–mortality
*w*/*v*	Weight over volume
